# A Hebbian Approach to Non-Spatial Prelinguistic Reasoning

**DOI:** 10.3390/brainsci12020281

**Published:** 2022-02-17

**Authors:** Fernando Aguilar-Canto, Hiram Calvo

**Affiliations:** Computational Cognitive Sciences Laboratory, Center for Computing Research, Instituto Politécnico Nacional, Mexico City 07738, Mexico; faguilarc2021@cic.ipn.mx

**Keywords:** Hebbian learning, BCM theory, Spike Timing-Dependent Plasticity, Temporal Difference Learning, Convolutional Neural Networks

## Abstract

This research integrates key concepts of Computational Neuroscience, including the Bienestock-CooperMunro (BCM) rule, Spike Timing-Dependent Plasticity Rules (STDP), and the Temporal Difference Learning algorithm, with an important structure of Deep Learning (Convolutional Networks) to create an architecture with the potential of replicating observations of some cognitive experiments (particularly, those that provided some basis for sequential reasoning) while sharing the advantages already achieved by the previous proposals. In particular, we present Ring Model B, which is capable of associating visual with auditory stimulus, performing sequential predictions, and predicting reward from experience. Despite its simplicity, we considered such abilities to be a first step towards the formulation of more general models of prelinguistic reasoning.

## 1. Introduction

In recent decades, a huge amount of research in Computational Neuroscience has resulted in advanced Hebbian learning rules, such as the Bienestock-Cooper-Munro (BCM) rule, the Spike Timing-Dependent Plasticity rules, and the Temporal Difference Learning algorithm. In addition, computer scientists formulated the Convolutional Neural Networks, which can be understood roughly as models inspired in some properties of the Visual Cortex but with great success in image classification tasks. Recent research studied the inclusion of Hebbian learning in Convolutional Networks to incorporate online learning in image classification tasks.

Our main goal is to explore the connection between existing computational models of neural networks and synaptic plasticity (learning) and the observable behavior in terms of pre-symbolic reasoning. More precisely, the main objective is to model a complete architecture of an artificial neural network capable of reproducing at least some of the experimental results on animal learning and reasoning by controlling only the external inputs rather than formulating a partial network with ideal characteristics. This aim requires a model of real-time perception since most experiments depend on the recognition of particular stimuli.

For this purpose, we will first introduce Hebbian-based rules in Section 1.1, then briefly discuss the neural-based concept of causality in Section 1.2. The use of Synaptic Timing-Dependent Plasticity as prelinguistic reasoning is introduced in Section 1.3. Related work that integrates both Computational Neuroscience models and Machine Learning (particularly Convolutional Networks) is presented in Section 2; particularly, preliminary work on Convolutional Neural Networks for modeling cognitive architectures is presented in Section 2.1. Theoretical foundations are detailed in Section 3: Firing rate rule (Section 3.1); Hebbian rule (Section 3.2); Temporal Difference Learning (Section 3.3); and details on the experiment of Sadacca et al., (2016) [1] (Section 3.4). Our proposed models and experiments related to this latter experiment are explained in Section 4. Results are presented in Section 5, followed by a short discussion in Section 6, and finally our conclusions are drawn in Section 7.

### 1.1. Hebbian-Based Rules

Since the experiments by Terje Lømo in 1966 and Timothy Bliss in 1973 [2,3], Hebbian-based rules have been empirically confirmed with the discovery of the process of Long-Term Potentiation (LTP) and Long-Term Depression (LTD). More specific reformulations of the classical Basic Hebb rule have emerged as the result of further research on synaptic plasticity, including the Covariance and Oja rules [4,5]. Some of the closest models with the biological experiments are the Bienestock-Cooper-Munro (BCM) and Synaptic Timing-Dependent Plasticity (STDP) learning rules.

BCM theory was proposed in the article of the referred authors in 1982 [6]. It can be considered to be a synaptic mechanism to avoid unbounded growth of the weights by adding a sliding threshold, which is an undesired (and unobserved) consequence of the Basic Hebb and Covariance learning rules [7]. The Oja rule is another possibility to control the weights. However, the BCM theory is more consistent with the recorded electrophysiological data than the previously proposed alternatives [8].

**Figure 1:**
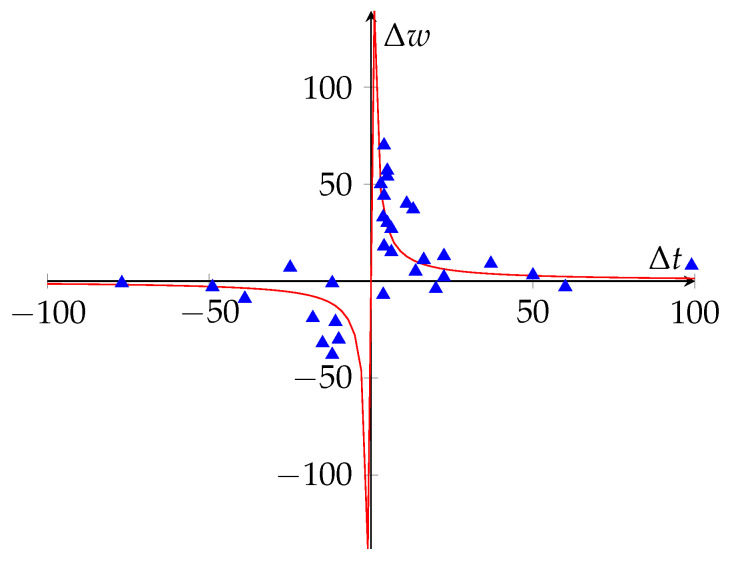
Percentual change in the amplitude of the Excitatory Postsynaptic Potential (EPSC) measured with the differente $t_{post}-t_{pre}$ (ms), according to the results of [9] (based on [4], redrawn using $H\left(\tau \right)=\frac{140}{\tau }$).

On the other hand, research conducted on frogs of the genus *Xenopus* showed the critical importance of the temporal difference between the activities of the post-synaptic and pre-synaptic neurons in the modulation of synaptic modification of the weights [9]. In this work, they noted that the activity (in terms of firing rate of action potentials) of the pre-synaptic neuron followed by the activity of the post-synaptic neuron results in LTP. However, if the post-synaptic activity precedes the pre-synaptic, it produces LTD. Moreover, if the difference between the times of both activities is small, the change of the synaptic modification increases (see Figure 1). The abovementioned results resulted in the formulation of the Synaptic Timing-Dependent Plasticity (STDP) rule.

### 1.2. On the Neural Basis of Causality

Different authors identified the STDP learning rule as the neural basis concept of causality [10,11,12]. Although the term of causality has been extensively discussed in other scientific disciples and Philosophy (see for example [13,14]), some aspects of what we conceive as causal inference might be captured by the STDP model. However, as we shall see, spurious correlations cannot be avoided with this paradigm. For instance, suppose that a hidden event A causes B and C with a short delay. An observer would see C after B, concluding that B causes C, which is not true. This problem is considered out of the scope of this article. Instead, we will consider STDP learning as one of the bases of sequential reasoning since it enables neurons to predict the following events based on the previous hypothesis (Markovian condition).

### 1.3. Prelinguistic Reasoning

Since STDP learning has been identified in animals such as amphibians and mammals, it can be considered to be a widely extended strategy for learning temporal correlations and a possible first stage of reasoning. Thus, it is relevant to observe how these mechanisms operate in non-human animals to create the so-called Proto-Logic [15] or pre-symbolic or prelinguistic reasoning.

In the context of artificial neural networks, prelinguistic reasoning is relevant to develop agents capable of taking decisions to achieve self-preservation, which is one of the possible main functions of this kind of reasoning. In this sense, Mercier and Sperber [16] state that complete logical reasoning is connected with argumentation and effective communication of ideas (a linguistic phenomenon), whereas prelinguistic reasoning is relevant in simple decision-making processes. Nevertheless, Proto-Logic can be much more complex than simple temporal-causal reasoning. According to Park [17], the formulation of a proper Proto-Logic might depend on (or include) a formulation of a spatial reasoning mechanism or *Proto-geometry*, which lies outside the scope of this work. Nevertheless, recent research was conducted to understand the neural basis of spatial cognition (see [18]).

## 2. Related Work

This research integrates two main concepts of Computational Neuroscience (BCM, STDP) and one related to Machine Learning (ConvNets, specifically Deep Learning) and another concept that originated as a Reinforcement Learning algorithm but nowadays is relevant in the field of Computational Neuroscience as a model of Dopamine reward prediction. This mixture is unusual in the literature, even though we can find works that try to understand the exact relationship between synaptic plasticity rules BCM and STDP [19,20], or include both rules in the same context [21,22]. Other papers try to integrate BCM, STDP, and Reinforcement Learning [23].

Papers that implement Hebbian-based rules in a typical Machine Learning context have also been published. In [24], BCM theory, Competitive Hebbian Learning, and Stochastic Gradient Descent are considered to derive a new learning rule. The integration of Hebbian-based learning with ConvNets has also been proposed [25,26,27,28], but BCM learning rules have been barely considered [29]. In addition, some of the previous works focused on improving the TDL algorithm, taking into account the results of [1], which includes the articles by [30,31,32,33].

Spiking Neural Networks (SNNs) are a bioinspired approach for neural networks, even though Deep SNNs have not yet achieved the results of deep Artificial Neural Networks (ANNs) [34]. STDP has been usually implemented in SNNs, in architectures such as a the neuromorphic SpiNNaker [35] or TrueNorth [36]. Other neuromorphic implementations of STDP were also proposed [37,38,39,40]. In the case of [41], the authors presented a deep convolutional network with STDP learning. Some properties of STDP in SNNs have been revealed, which is the case of [42], showing the emergence of Bayesian computation with STDP. One remarkable application of STDP in a Machine Learning problem was achieved by [43], reaching an accuracy of 95 % in classification of the dataset MNIST. Moreover, it was an attempt to understand the Backpropagation algorithm with STDP theory [44]. For a full review of different applications of SNNs with Hebbian-based rules, including STDP and BCM, see [45].

### 2.1. Preliminary Work

In [29], a neural architecture with a convolutional network was proposed. The convolutional network with pre-trained weights operates as a feature extractor. A final layer with Hebbian learning enables performing real-time learning for image classification. This network can be used to teach the system to discriminate visual stimuli. The usage of Convolutional Neural Networks (ConvNets) in cognitive architectures is controversial for some authors, as some researchers (such as [46]) do not consider them as proper models of Visual Cortex. However, the models of ConvNets capture some of the basic principles of the Hierarchical Model of visual perception. In addition, ConvNets have achieved great success on large image recognition tasks. Moreover, ConvNets are the best model to explain the neural representations of the Inferior Temporal cortex [47], which have been labeled as the place where complex visual recognition occurs. For some authors, such as [48], these results show that it is possible to admit deep neural networks are cognitive models. In our case, we will state that our attempt tries to propose an artificial architecture able to simulate some cognitive experiments, while the search for more bio-inspired systems is an ambition that might not be reached yet.

### 2.2. Experimental Results on Animal Learning

One astonishing advance in the field of computational cognitive sciences was the development of the Temporal Difference Learning (TDL) algorithm as well as its interpretation as a model of the Dopamine Reward System [49,50,51]. This model is particularly good for our purposes because it provides an explicit mechanism of prediction of reward, which is relevant in the context of Reinforcement Learning. Nevertheless, the experiments performed in rats by [1] showed some of the limits of the TDL method, by showing that some inferences do not require previous experience.

A more specific goal for this work consists of integrating the computational models of ConvNets and TDL with the BCM and STDP learning rules to develop an architecture that emulates *grosso modo* the observations of articles such as [1]. This system might not only be able to learn to differentiate complex visual stimuli but also to perform inferences with the learned stimuli and (artificial) rewards. More details of the work of Sadacca et al., (2016) will be given in Section 3.4.

## 3. Theoretical Background

### 3.1. Firing Rate Model

First of all, it is necessary to define the basic model of neural activity that will be used throughout the text. The election of the model of neural dynamics is highly motivated according to the purposes of the research. In this case, we require an extremely efficient model due to resources limitation for further implementation. One plausible possibility (despite its simplicity) is the linearized firing rate model, which represents each activity as the frequency of spikes (action potentials) per second, measured in hertz (Hz). In this manner, the activity of an individual neuron is given by v=f(w·u) where $u=(u_{1},\cdots ,u_{m})$ represents the activities of presynaptic neurons, $w=(w_{1},\cdots ,w_{m})$ the respective connection weights and $f:{\mathbb{R}}^{+}\to {\mathbb{R}}^{+}$ is an activation function.

### 3.2. Hebbian Rules

As stated in the introduction, several Hebbian-based rules have been discussed in the literature, including the BCM and STDP paradigms. Here we will discuss three important plasticity rules, which will be used in this article.

#### 3.2.1. The Oja Rule

The Oja rule [5] is a modification of the Basic Hebb rule with the addition of a penalty on the factor $v^{2}w$ to impose a dynamic constrain on the sum of the squares of the weights [4]. Hence, the Oja rule is given by

(1) $$\tau_{w}\frac{dw}{dt}=vs.u-\beta v^{2}w.$$

#### 3.2.2. The BCM Rule

The BCM learning rule [6] was proposed in the same year of the Oja rule (1982), and it is another manner to control the growth of the weights by introducing a dynamic threshold θ which decides whether the activities produce potentiation or depression. Thus, the BCM rule is given by a couple of differential equations [7]

(2) $$\begin{array}{cc} \tau_{w}\frac{dw}{dt} & =vs.u(v-\theta ), \end{array}$$

(3) $$\begin{array}{cc} \tau_{\theta }\frac{d\theta }{dt} & =v^{2}-\theta , \end{array}$$

where $\tau_{w}>0$. The BCM rule is often considered to be a Spike Rate-Dependent Plasticity rule, which includes other learning rules such as Basic Hebb, Covariance, and Oja.

#### 3.2.3. Spike Timing-Dependent Plasticity Rules

The Spike Timing-Dependent Plasticity (STDP) rules appear as a formalization of the experiments reported in the seminal papers of [9,52]. In the following years, STDP was observed in a wide variety of organisms, ranging from insects to humans [53]. A model of the dynamics of the weights is given by the following Equation [4]:

(4) $$\tau_{w}\frac{dw}{dt}=\int_{0}^{\infty }\left(H(\tau )v(t)u(t-\tau )+H(-\tau )v(t-\tau )u(t)\right)d\tau .$$

H(τ) represents the function of the temporal window that models the behavior of Δt respecting Δw. For instance, in Figure 1, $H\left(\tau \right)=\frac{140}{\tau }$ is a possible candidate to model the recorded data. In Equation (4), note that if *H* satisfies H(−τ)=−H(τ) and the sign of H(τ) is the sign of τ, the term H(τ)v(t)u(t−τ) can be understood as the LTP quantity whereas H(−τ)v(t−τ)u(t)=−H(τ)v(t−τ)u(t) represents the LTD term. As shown in [53], there is a diversity of *H* windows. Such variety can be related to different purposes, as we shall discuss.

Assuming that $H\left(\tau \right)=\frac{a}{\tau }$, we can perform an extreme discretization of Equation (4) in the following manner:

$$\begin{array}{cc} \tau_{w}\frac{dw}{dt} & =\int_{0}^{\infty }\left(H(\tau )v(t)u(t-\tau )+H(-\tau )v(t-\tau )u(t)\right)d\tau ,\\ \; & =a\int_{0}^{\infty }\left(\frac{1}{\tau }v\left(t\right)u\left(t-\tau \right)-\frac{1}{\tau }v\left(t-\tau \right)u\left(t\right)\right)d\tau ,\\ \; & =a\lim_{b\to \infty }\lim_{n\to \infty }\frac{b}{n}\sum_{k=1}^{n}\left(\frac{1}{\tau_{k}}v\left(t\right)u\left(t-\tau_{k}\right)-\frac{1}{\tau_{k}}v\left(t-\tau_{k}\right)u\left(t\right)\right) \end{array}$$

The fact that only response in a time interval of 50 ms is another further consideration of STDP, which means that $\lim_{\tau \to \pm \infty }H\left(\tau \right)=0$. It also holds for the selected *H*. Therefore, an extreme simplification only considers the first term, leading to this reduced rule:

(5) $$\tau_{w}\frac{dw}{dt}=\left(v(t)u(t-1)-v(t-1)u(t)\right).$$

**Figure 2:**
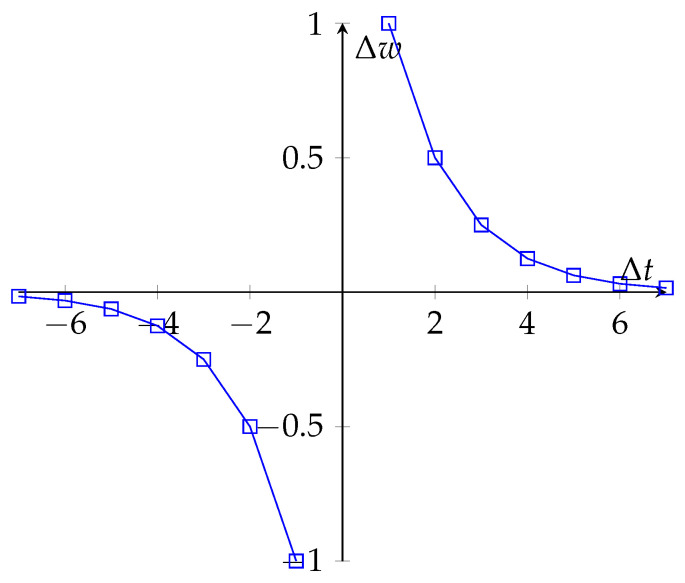
Simulated behavior of the simplified model with a recurrent self-connection $w_{r}=0.5$ and h=(1,−1).

We can generalize the previous expression to the following equation:

(6) $$\tau_{w}\frac{dw}{dt}=\left(v\left(t\right)u\left(t\right),v\left(t-1\right)u\left(t\right)\right)\cdot h$$

where $h\in {\left\{-1,0,1\right\}}^{2}$ represents a vector that simplifies function H(τ). This reduction might be considered extreme, but it might be possible to approximate the observed behavior using other neural mechanisms that prolong a signal, see Figure 2. Nevertheless, this observation is a curiosity of the simplified model, and what exactly occurs in biological neurons should be discovered and confirmed by experiments.

### 3.3. Temporal Difference Learning

**Figure 3:**
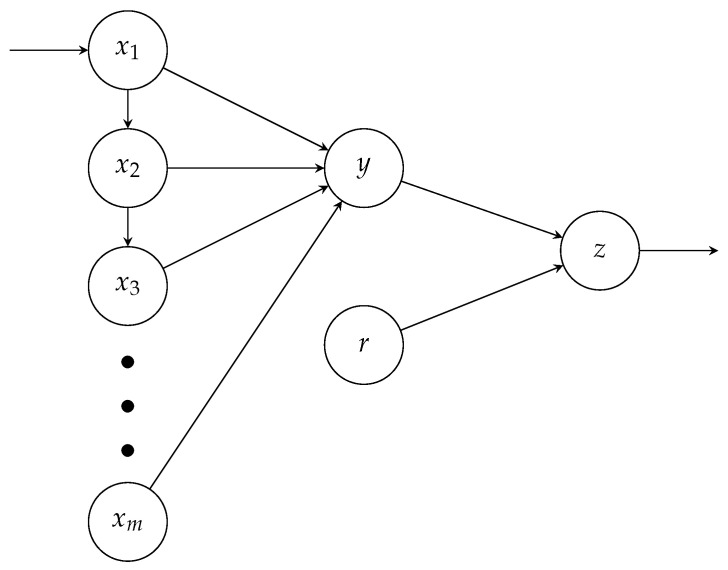
Architecture for TDL based on [54].

Recurrent self-connections and Hebbian rules can be useful to associate a reward with a given stimulus, even if it is presented a few seconds after the target associated object. However, strong evidence supports that the mechanism underlying the reward prediction system of dopaminergic neurons is close to the Temporal Difference Learning algorithm. Our description of the TDL is a slight variation of the neural network model described in [54]. Let us consider the neural network provided in Figure 3, where $x_{1},\cdots ,x_{m}$ are temporal neurons with connections $w_{x_{j},x_{j-1}}=1$ (which yields $x_{j+1}\left(t+1\right)=x_{j}\left(r\right)$), *y* the activity of the “collector neuron”, *r* the activity of the reward neuron (r=1 if a direct and clear reward is presented) and *z* the activity of the dopaminergic neuron: z(t)=1 if a reward is predicted and can be associated with a decision-making process.

The activity of the collector neuron *y* is given by the following equation:

(7) $$y\left(t\right)=\sum_{j}w_{j}\left(x_{j}\left(t\right)-x_{j}\left(t-1\right)\right),$$

and the weights $w_{z,y},w_{z,r}$ are set to 1. Thus,

(8) z(t)=r(t)+y(t).

Finally, the learning rule of the TDL algorithm is quite different from Hebbian learning rules and is given by

(9) $$w_{j}\left(t+1\right)=w_{j}\left(t\right)+\alpha \;\mathrm{DA}\;x_{j}\left(t-1\right),$$

where DA=z(t) and α>0 is the learning rate. Hence,

(10) $$w_{j}\left(t+1\right)=w_{j}\left(t\right)+\alpha x_{j}\left(t-1\right)z\left(t\right).$$

### 3.4. The Experiment of Sadacca et al., 2016

A remarkable experiment conducted by [1] in rats showed the limitations of Temporal Difference Learning as the sole algorithm that models the activity of neurons of the Midbrain structures (such as the Ventral Tegmental Area, VTA). This research is particularly interesting for our purposes because it reviews the relationship between temporal reasoning and reinforcement learning with reward. As noticed by [55], not all the predictions were carried out, but dopaminergic neurons depend on previous experience.

In general terms, Sadacca et al. designed the experiment with three principal stages: preconditioning, conditioning, and probe test. As subjects of the experiments, they selected 14 adult Long-Evans rats. In the preconditioning phase, rats received four auditive stimuli (A, B, C, and D) presented in pairs: A–B and C–D appeared sequentially without delay. Each stimulus lasted 10 s with 3–6 min of separation per trial. Overall, this stage took two days with a total of 12 trials.

Once the rats completed the preconditioning phase, they started the conditioning stage for six days. In a single day, rats received stimulus B six times followed by a liquid reward in a lapsus of 1–7 s, consisting of flavored milk. In addition, they received stimulus D without any reward. Once again, each stimulus appeared 10 s, and the inter-trial period lasted 3–6 min.

Finally, rats received a probe test, consisting of the presentation of stimuli A and C without reward, to measure the activity of dopaminergic neurons related to reward prediction. In addition, researchers provided three reminded presentations of stimulus B followed by a reward and three trials of stimulus D without reward.

As expected, when researchers measured the reward prediction when B or D were presented, stimulus B showed a stronger association with the reward. Rats also showed a higher response when stimulus A appeared than clue C. During the experiment, the authors identified three types of neurons: putative GABAergic, putative dopaminergic, and unknown type (inhibitory to stimulus and rewards). In this context, GABAergic cells usually fire when they identify the presence of reward, the putative dopaminergic are associated with reward prediction, whereas the non-classified neurons are inhibitory to stimulus and rewards. Although dopaminergic cells responded strongly to stimuli A and C, they showed a significant preference for A. Researchers interpreted the response for C as saliency or novelty, but in the case of A, it is difficult to explain this preference solely based on the mentioned criteria.

To summarize, this experiment reveals a transitive property of reinforcement learning in the brain: if A precedes B, C precedes D, and B predicts reward whereas D does not, we can conclude that A predicts reward but C does not. This process might be one of the neural bases of inference rules and can reveal how prelinguistic reasoning is organized at the cellular level.

The experiment of [1] might be considered to be a continuation of the previous work of [56], as it follows the same methodological design. In the case of [56], the authors achieved similar findings in the Orbitofrontal Cortex (OFC) and highlighted the relevance of this cerebral region in the sequential inferences. As [55] points out, the TDL is unable to predict reward in unobserved circumstances, at least in the classical formulation. Gradually, these findings and others support the idea that reinforcement learning carried out in the neural systems is model-based rather than model-free (at least in complex nervous systems), as it was originally formulated in Temporal Difference Algorithm.

## 4. Materials and Methods

In this section, we present two models to develop an architecture able to be tested with the experiment of [1]. Both models have the same basic structure, but they differ in key elements. Temporal Difference Learning is not included in Model A, which only operates with associations. Another main difference is the usage of the BCM learning rule in Model B for visual learning tasks, whereas Model A uses the Oja rule instead.

### 4.1. Experimental Description

Instead of testing an artificial network model with a given dataset with rigidly defined training, validations, and testing sets, this research aims to evaluate the architecture differently, most closely to treat the system’s behavior as a separate organism and describe the properties of the architecture. In other words, it means that experiments conducted on animals need to be replicated in a computational context, and the values of the artificial neural activity can be recorded by tracking the output values of individual neurons.

The experiment will follow the general lines of the steps established in [1], with some modifications. The system’s inputs are a camera (integrated webcam) and a keyboard. For instance, a reward is delivered when the key r is pressed. The system only prints some results as an output, but a Text-to-Speech system was included.

1. Four visual stimuli A, B, C, and D are presented. The selected stimulus are the following:
  A Potato or lemon.
  B Medicine tablet.
  C Silver coin.
  D Notebook.
  In this stage, the system needs to learn to discriminate the stimuli by labeling the visual pattern with the linguistic description (name).
2. The stimuli are presented during 10 s, and then, A, B, and C, D are presented sequentially without delay, as the pre-conditioning stage. Each trial is separated with intervals of more than 30 s. This procedure is replicated 6–7 times.
3. The final step corresponds to the conditioning stage: stimulus B is presented during 10 s and after 1, 4, and 7 s an artificial reward is presented during a group of 3–9 trials. D is presented during 10 s without reward. Each operation is separated in an interval of more than 30 s.

There are some slight differences from the original methodology illustrated by [1]. First, the stimuli selected were auditory and not visual. This election might have been driven by the great capacity of auditory recognition on the rat’s brains since their auditory cortex is larger than their visual cortex. In our case, as we describe later in the models’ sections, we do have an architecture capable of learning visual recognition in real time; however, we lack the required structure for auditory recognition. Future research on this topic might fill this gap. Another difference is the timing used for each stage. The intervals between trials are separated with intervals of 3–6 min in the original experiment, whereas in this case, we only use 30–60 s. This reduction was performed to prevent a memory leak.

### 4.2. Recurrent Network with STDP Learning

Spike Rate and Spike Timing-Dependent Plasticity might be used with different purposes in a diversity of plasticity learning rules. In this article, the Spike Rate-Dependent rules (Oja and BCM) will be used in a different context, but as we will see, the STDP rule can be effectively used for temporal inferences. In this sense, we will use the notation A↠B as a temporal succession of event *B* given *A*. This notation should not be confused as the logic form of *A implies B* or the close concept of causality *A causes B* since a third element could be involved (see example in Section 1.1).

A more formal definition would state that A↠B (B precedes A) if and only if *B* occurs within an interval $\left[T_{1},T_{2}\right]$ ($T_{1}>0$) after the occurrence of *A*. For instance, if *B* happens three seconds after *A*, the definition holds. Now, we need to construct a recurrent neural network that can perform these temporal inferences. Let $r=(r_{1},\ldots ,r_{K})$ neurons that selectively fire when a specific object is presented. Highly selectively neurons firing from objects have been found in the Inferior Temporal cortex [57]. If *B* precedes *A* and *A* do not precede *B* in any case, we say that *B* precedes *A* exclusively (A↣B).

Temporal inferences can be learned with a full connectivity matrix $S\in R^{K\times K}$ as the weight matrix of a Elman-type recurrent network and STDP learning in all connections excluding the self-recurrent links. In the matrix, this means that ${\left[S\right]}_{ii}=\lambda_{i}\in \left[0,1\right)$ and for simplicity, let $\lambda_{i}=\lambda$. If $r_{i}\left(t\right)=1$ for $t\in [T_{1},T_{2}]$, then $r_{i}\left(t+\tau \right)=\lambda^{\tau }$, allowing keeping the neuron firing a few seconds after the presence of the stimuli.

The activity of the network is given by

(11) $$r\left(t+1\right)=\sigma_{l}\left(\kappa \left(S\right)r\left(t\right)+f\left(v\left(t\right)\right)\right),$$

where v(t) is the input of the network, typically the output of recognized items of a convolutional network. An activation function $f:R^{+}\to \left[0,1\right]$ is needed because we require to interpret a value as presence or absence of a particular stimuli. The activation function $\sigma_{l}$ is

(12) $$\sigma_{l}\left(x\right)=\left\{\begin{array}{cc} 1 & x>1\\ x & x\in [l,1]\\ 0 & x<l \end{array}\right..$$

We set parameter *l* to 0.1 in all the experiments. In addition, κ is a control function of the weights, and it is given by

(13) $$\kappa \left({\left[W\right]}_{ij}\right)=\sigma_{0}\left(W_{ij}\right).$$

With this construction, and assuming an ideal scenario, we can prove that forward inference is possible.

**Proposition** **1.** *Let S the connectivity matrix with STDP learning of a fully recurrent network. Let $r_{i}=1$ if and only if stimuli A is presented and $r_{j}=1$ if and only if stimuli B is presented.*

1. *Let us consider h=(1,0). If A↠B and $r_{i}\left(t\right)=1$, then $r_{j}\left(t+1\right)=1$, assuming a sufficient number of presentations of A.*
2. *Let us consider h=(1,−1). If A↣B and $r_{i}\left(t\right)=1$, then $r_{j}\left(t+1\right)=1$, assuming a sufficient and non-vanishing number of presentations of A.*

**Proof.** 

1. If A↠B, therefore each presentation of *A* is followed by a presentation of *B*. Then, if $r_{i}\left(t_{k}\right)=1$, $r_{j}\left(t_{k}+\tau_{k}\right)=1$. In $t=t_{k}+\tau_{k}$, $x_{i}\left(t_{k}+\tau_{k}\right)=\lambda^{\tau_{k}}$, which means that
  $$\begin{array}{cc} \frac{dw_{ji}}{dt} & =\frac{1}{\tau_{w}}r_{i}\left(t\right)r_{j}\left(t-1\right)\\ \; & >\frac{1}{\tau_{w}}\lambda^{\tau_{k}}\\ \; & \geq \frac{1}{\tau_{w}}\lambda^{T_{2}} \end{array}$$
  Applying this update to $w_{ji}$ several times yields:
  (14) $$w_{ji}>\sum_{k}\frac{1}{\tau_{w}}\lambda^{T_{2}}.$$
  Enough presentations yield $w_{ji}\geq 1$. Thus, if $r_{i}\left(t\right)=1$, then $r_{j}\left(t+1\right)\geq \sigma_{l}\left(\kappa \left(w_{ji}\right)r_{i}\left(t\right)\right)=1$.
2. If A↣B, therefore A↠B and LTD does not occur. Thus, using a similar argument of item 1 yields the result.
   □

### 4.3. Ring Model A

As mentioned before, the construction of a full computational model demands the integration of a sensory system that complements the partial mechanism of the Recurrent Neural Network with STDP learning. The general scheme includes the inclusion of a Convolutional Network to extract image features, one additional layer fully connected with the feature vector and Spike rate-based learning, the recurrent network with STDP learning (the *Ring*), and a final mechanism for reward prediction. A Speech-To-Text (STT) system is used to input voice in order to associate a word with a new visual stimulus.

The first part of the model (sensory inputs) is based in the architecture developed in [29]. Based on these results, the Xception network [58] was used for feature extraction and the Oja learning rule as the model of synaptic plasticity. In formal terms, let $u\in R^{\ell }$ the feature vector extracted with the ConvNet (which means, if *I* is the image, then u=ConvNet(I)). Let us consider the classification vector for *K* classes $v=\left(v_{1},\cdots ,v_{K}\right)\in R^{K}$, such that $v_{i}=1$ if the auditory pattern *i*-th is recognized by STT (if the pattern is not in the database, it adds the pattern to an empty entry of **v**). Let H be the weight matrix. Then, v=Hu, following the linear version of the firing rate model. It is worth mentioning that all Hebbian matrices are initialized with zeroes.

**Figure 4:**
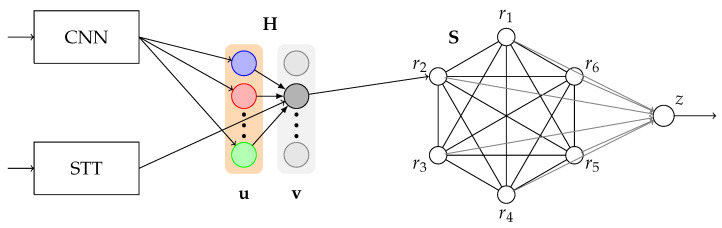
Schematic representation of Ring Model A. For visual simplicity, some connections are not presented, such as the recurrent self-connections of r. The feature vector u is fully connected with the Hebbian layer v, but the diagram is focused on the second recognized item. Each entry of v is connected with one entry of r, as well. Additionally, K=6 in this particular case.

Finally, a neuron with activity *z* is connected with each neuron $r_{i}$ and with itself forming a recurrent connection with fixed $w_{zz}=\lambda$. The rest of the weights learn via STDP. z(t)=1 when a reward is presented or when a reward is predicted. A full representation of this model is provided in Figure 4.

### 4.4. Ring Model B

**Figure 5:**
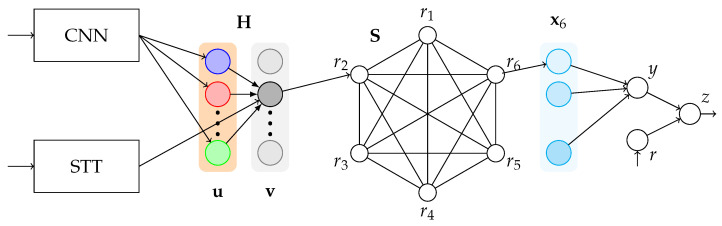
Schematic representation of Ring Model B. For visual simplicity, some connections are not drawn (see the caption of Figure 4). In addition, $x_{i}$ por i=1,…,5 are not visible.

Model B follows the basic structure of Model A with several improvements. Instead of the Oja rule, it implements the BCM rule in the weight matrix H. Another major modification is the usage of Temporal Difference Learning instead of a single neuron to perform reward prediction. Each value $r_{i}$ is connected with a temporal vector $x_{i}=\left(x_{i,1},\ldots ,x_{i,T}\right)$, such that $x_{i,1}=r_{i}$. Finally, each temporal vector is connected to the collector neuron *y*. *z* and *r* follows the architecture of TDL described in their respective subsection. In addition, in order to enhance the results on image classification, the input image is centered and fixed with 299×299 pixels. This model is shown in Figure 5.

## 5. Results

According to Proposition 1, given some ideal conditions, we can perform some forward inferences only by applying the Recurrent structure (Ring). The real challenge consists in trying to adequate all the elements to observe this idealistic scenario. Both models A and B are capable of replicating the experiment of [1], however, since model A does not include TDL, it is not possible to disassociate a learned reward, which is an important property observed in Midbrain dopaminergic neurons.

### 5.1. Real-Time Learning in Image Classification

Online learning is one of the main advantages of the Hebbian approach in contrast with classical gradient-based optimizers. The comparison performed in [29] shows that Hebbian methods such as Basic Hebb, Covariance, and Oja rules with convolutional support are almost able to reach the accuracy of gradient-based optimization, including relatively recent optimizers such as Adam or RMSprop. One additional difficulty in this specific context relies on the necessity of using an activation function to map the output of the Hebbian layer to the set [0,1]. In this case, the activation function was the Heaviside step function with threshold $\theta_{H}$. Nevertheless, the selection of threshold was challenging.

One principal difference between models A and B is the usage of the Oja and BCM learning rules to train the weight matrix H. The BCM rule did not show good results in image recognition in [29]. Nevertheless, the original implementation keeps θ fixed to 1, whereas in this case it was set as dynamic.

To test both learning rules, we defined the following procedure to evaluate the first stage of the main experiment:

1. Delay 10 s.
2. Show the item to the camera and input audio with the name of the item (training step).
3. Hold the item for 10 s.
4. Retire the item and wait 10 s.
5. Show the item and hold it during 10 s.
6. Repeat step 2 *q* times.

**Figure 6:**
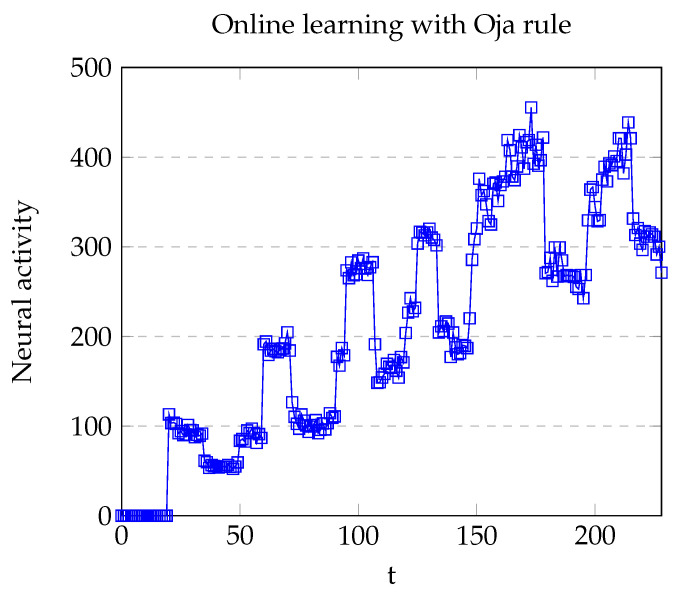
Plot of the neural activity of the neuron $v_{1}$ with the Oja learning rule. Local maxima (upper peaks) appeared when the pattern was presented, whereas the local minima appeared in absence of the pattern. Abrupt increments in the neural activity were due to the enhancement of the weights via audio.

The item shown in both models was the silver coin. In Model A (Oja), q=6. Figure 6 depicts the change of activity of a particular neuron $v_{1}$ (or more generally $v_{A}$) associated with the presence or absence of stimulus *A*. As can be seen, the learning method complicates the election of a particular threshold $\theta_{H}$. Another problem (arguably worse) is that more than one training step yields the necessity of using different thresholds for each neuron $v_{i}$. For that reason, only one training step was used in the Model A. These referred problems are even worse in Basic Hebb and Covariance learning rules, since at least the Oja rule imposes a regularization in their weights.

**Figure 7:**
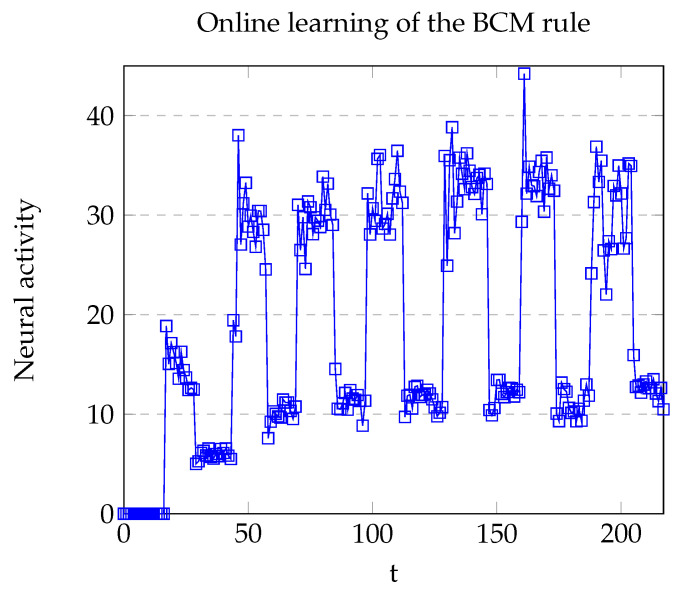
Plot of the neural activity of the neuron $v_{1}$ with BCM learning rule. Local maxima (upper peaks) appeared when the pattern was presented, whereas the local minima appeared in absence of the pattern. Abrupt increments on the neural activity were due to the enhancement of the weights via audio.

The mentioned problems are mostly solved with the inclusion of the BCM rule. As shown in Figure 7, new training steps do not affect the existence of an implicit margin between absence or presence of the labeled stimulus.

### 5.2. Ring Model A

**Figure 8:**
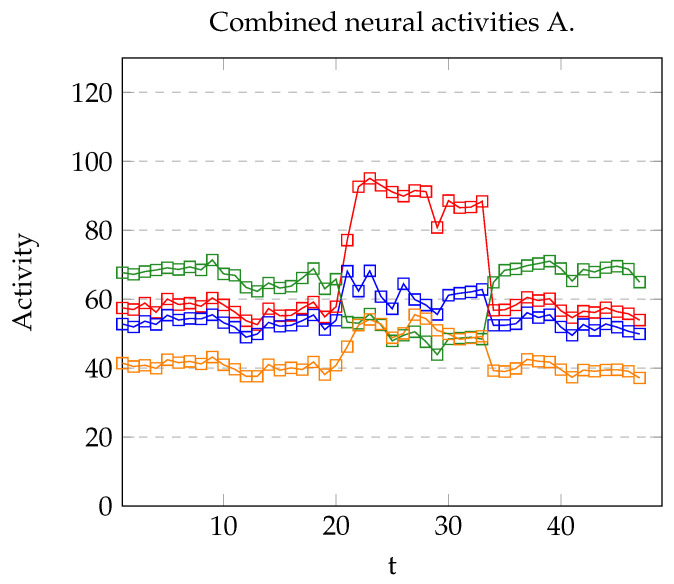
Neural activity of $v_{A}$ (blue), $v_{B}$ (red), $v_{C}$ (green), and $v_{D}$ (yellow) using Model A. Stimulus B was presented in the time interval [21,33].

**Figure 9:**
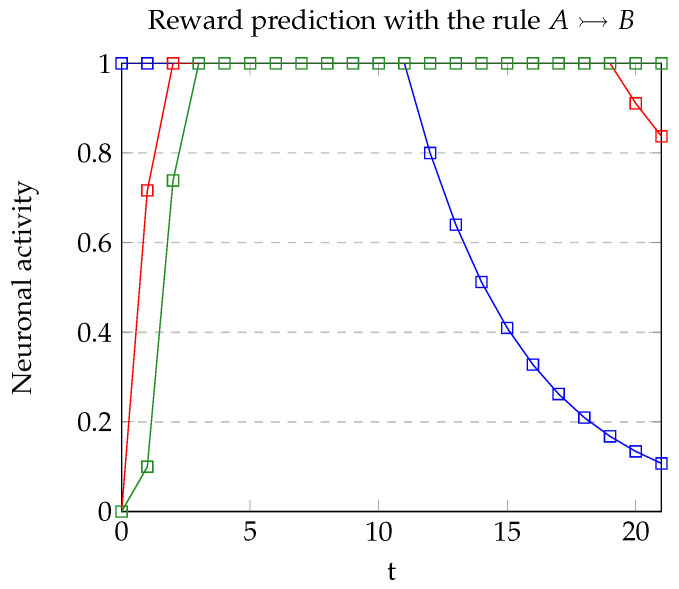
Neural activity of $r_{A}$ (blue), $r_{B}$ (red) and *z* (green).

As indicated previously, model A was able to repeat the results of the target experiment on at least one occasion. Nevertheless, some trials were discarded since an incorrect recognition resulted in an incorrect association in the recurrent structure. This situation is due to the low margin between the recognized presence of a stimulus and its absence (see Figure 8). In the successful completion of the experiments, the reward was correctly predicted. As Figure 9 shows, when stimulus *A* is presented $r_{A}=1$, and in the next two iterations, $r_{B}=1$, allowing $r_{z}$ to increase until z=1.

### 5.3. Ring Model B

**Figure 10:**
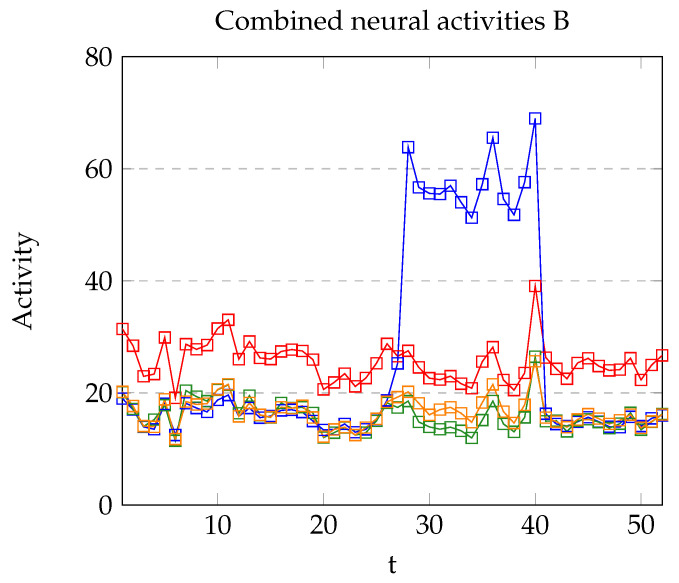
Neural activity of $v_{A}$ (blue), $v_{B}$ (red), $v_{C}$ (green), and $v_{D}$ (yellow) using Model B. Stimulus A was presented in the interval [28,40].

Model B shares most of the features achieved in the original Model A, but it solves some of the related problems within it. As shown in Figure 10, the margin between a presented stimulus and absent is stronger. Five training steps were used in this case, which is an advantage compared with the one training step of Model A. Nevertheless, the pre-conditioning phase required seven iterations (instead of 6), and the conditioning stage needed nine iterations (instead of 3) to reach the value z≥1 and execute the output. By increasing the learning rate this situation might be improved.

## 6. Discussion

Integration of the key concepts taken from Computational Neuroscience and Deep Learning to generate complete cognitive architectures is still a challenge for computational and mathematical modeling. In this approach, we have focused on the prelinguistic and non-spatial structures related to two principles of reasoning: the relationship A↣B (encoded by STDP learning) and the transitive property in reward prediction (described by experiments such as [1]). We designed our system following the proposed design, and it verifies both principles of non-spatial reasoning.

Some elements of the proposed models (in particular Ring Model B) have some functional parallels (at least slightly) with specific brain areas. As discussed, the ConvNet is somehow inspired in the models of the dorsal stream of Visual Cortex, which are areas V1, V2, and V4. The final feature vector can be associated with the Inferior Temporal area [47]. The SST system is not a model of Auditory Cortex but it performs a similar function. In the Ring Model B, the Temporal Difference network is inspired on the dopaminergic neurons of the Ventral Tegmental Area (VTA). Finally, the research [59] seems to relate the Orbitofrontal Cortex (OFC) with the inferences required to complete the reward prediction, carried out in the VTA. In our case, the Ring is useful for this purpose. Although more biologically inspired networks are needed to improve this model, this implementation might be useful for further changes.

The term “non-spatial prelinguistic reasoning” seems to involve temporal reasoning. Nevertheless, the proposed models did not cover some properties of this type of reasoning yet. Recent research has shown the existence of Time cells [60], which are elements of the Temporal Difference Algorithm. Another aspect that might play a role in the temporal inferences is the phenomenon of Phase precession [61], which is also related to the activity of time cells. Moreover, some authors have highlighted how Phase precession can facilitate STDP [62]. Nevertheless, it is important to emphasize that Time cells are also Place cells [63], and thus, we cannot dissociate temporal reasoning from spatial reasoning.

Finally, despite the referred limitations, the model can be gradually improved to cover other details of reasoning and cognition, including Place cells and other hippocampal neural systems. Representation in SNNs is also possible since the convolutional structure might be replaced by Deep SNNs adapted for object classification (such as the architecture provided by [41]). This change might be ideal since phenomena such as Phase precession are better described with spikes rather than in terms of the continuous firing rate. It also can help to present hardware implementations with the aid of memristors (see [40]) or other neuromorphic architectures (see [37,38]).

## 7. Conclusions

This research aimed to start using the cumulative knowledge (BCM theory, STDP, TDL algorithm, deep networks) acquired during recent decades to replicate cognitive experiments originally tested on animals. In this sense, evaluation of Artificial Intelligence bioinspired algorithms can be tested directly as independent cognitive entities (such as animals), which can be complemented with the classical evaluation metrics such as the accuracy on the testing set of a given dataset. Both are different challenges, in particular, this approach needs to operate in real time, and therefore, the learning algorithms should be online, which is a new complication. However, some of the discussed methods have been tested with classical methods, such as the visual recognition algorithm in [29], whereas TDL, BCM, and STDP have been directly contrasted with the biological experiments.

The neural network architectures proposed in this article expand the capabilities of the original model introduced in [29], and therefore this work is a direct continuation of previous work, which discusses the possibility of using Hebbian learning in an object classification context. This preliminary work provides the necessary tools to process the visual stimuli and connect in a proper architecture to perform the forward inferences.

In Proposition 1, it is shown that the Ring Model (the recurrent network) can learn sequential rules such as A↠B and A↣B. With the addition of Temporal Difference Learning, this system has expanded capabilities, and it was possible to replicate the observations of [1], which was the main objective of this research. This approach is the first step towards a general model of reward-oriented reasoning since the forward inferences analyzed are temporal versions of the syllogism *Modus Ponens*, which can be the most basic form of Logic shared by most animals.

Nevertheless, our main objective not only consists of simulating computationally one of the several cognitive experiments on mammals. Both Ring Models share the advantages of the previous research, including the possibility of real-time learning of object classification. In the case of Ring Model B, Temporal Difference Learning was included, which enhances the association of stimulus with rewards, and weak the connections if the reward is no longer presented.

### 7.1. Limitations

Like the rest of the convolutional networks, one limitation of our proposal relies on its simplicity, despite the advantage it represents. However, the main functional difference with the original experiment might be the lack of an unsupervised learning algorithm, since the rats were not conditioned to label auditory and visual information. In terms of implementation, the aid of parallel processors, memristors, or other neuromorphic architecture might improve this work since all the computations were presented sequentially.

Additionally, other concepts from Computational Neuroscience can complement this preliminary work, including the effects of Phase precession, properties of Time cells, and the relationship with Spatial reasoning. The Markovian property of the Recurrent Network (the Ring) seems to be another limitation. Perhaps by considering the phenomenon of Phase precession, we can add non-Markovian inferences to our mode.

### 7.2. Further Research

The future line of research the authors expect is the inclusion of other cognitive experiments, susceptible to be modeled with the current ideas of Computational Neuroscience. This idea might gradually expand the capabilities of the network. In addition, it is worth mentioning that the Ring Model B has potentially many more neurons than Model A since it has KT+2 neurons, which could be considered inefficient for large values of *K* or *T*. This problem might be discussed in the future. Moreover, it is relevant to say that most of the effort invested in this research was used to develop a proper architecture that might be used as the basis of future cognitive architecture, able to be implemented in a device such as a robot.

## Data Availability

Data and code can be found at https://github.com/Pherjev/TempoHebbian (accessed on 14 February 2022).

## Abbreviations

The following abbreviations are used in this manuscript:

BCM	Bienestock-Cooper-Munro
STDP	Synaptic Timing-Dependent Plasticity
TDL	Temporal Difference Learning
LTP	Long Term-Potentiation
LTD	Long Term-Depression
ConvNet	Convolutional Network
CNN	Convolutional Neural Network (alternative form)
MNIST	Modified National Institute of Standards and Technology (dataset)
STT	Speech-To-Text
TTS	Text-To-Speech
VTA	Ventral Tegmental Area
OFC	Orbitofrontal Cortex
